# Sex-Specific Association Patterns of Bone Microstructure and Lower Leg Arterial Calcification

**DOI:** 10.1007/s00223-024-01299-w

**Published:** 2024-10-14

**Authors:** Mikolaj Bartosik, Alexander Simon, Björn Busse, Florian Barvencik, Michael Amling, Ralf Oheim, Felix N. von Brackel

**Affiliations:** 1 Department of Osteology and Biomechanics, University Medical Center Hamburg-Eppendorf, Lottestr. 59, 22529 Hamburg, Germany; 2 Division of Orthopaedics, Department of Trauma and Orthopaedic Surgery, University Medical Center Hamburg-Eppendorf, Hamburg, Germany; 3 Interdisciplinary Competence Center for Interface Research (ICCIR), Hamburg, Germany

**Keywords:** Atherosclerosis, Vascular calcification, Lower leg arterial calcification (LLAC), Osteoporosis, Bone microstructure, High-resolution peripheral quantitative computed tomography, HR-pQCT

## Abstract

In conversations about bone loss and the importance of calcium homeostasis, patients frequently inquire about the association with arterial calcifications. Although a relationship between bone loss and the occurrence of vascular calcifications is suspected, it is not yet fully investigated and understood. This study aims to analyze associations between bone mineralization, structure, and vascular calcification at the lower leg in patients with low bone mineral density in HR-pQCT. We retrospectively analyzed 774 high-resolution quantitative computed tomography (HR-pQCT) scans of the distal tibia for the presence of vascular calcifications. After sex-specific propensity score matching for age and BMI to account for confounders, 132 patients remained for quantification of bone microstructure, bone density, lower leg arterial calcification (LLAC), and laboratory parameters of bone turnover. The interactions between bone parameters and vascular calcification were quantified by regression analyses. The calcium metabolism was not different between individuals with and without LLAC, nor oral calcium supplementation. Female patients with LLAC had a higher cortical perimeter (*p* = 0.016) compared to female patients without LLAC, whereas male patients with LLAC had lower cortical pore diameter than male patients without LLAC (*p* = 0.027). The appearance of LLAC was sex specifically associated with bone parameters. In female patients, only plaque density was associated with HR-pQCT bone parameters and age, whereas in male patients, plaque volume was associated with HR-pQCT parameters of the distal tibia. Female patients exhibit an increasing plaque density depended on age and trabecular thinning. Decreasing cortical pore diameter and trabecular number along with increasing bone mineralization are linked to increasing plaque volume in male patients.

**Supplementary Information:**

The online version contains supplementary material available at 10.1007/s00223-024-01299-w.

## Introduction

**Fig. 1:**
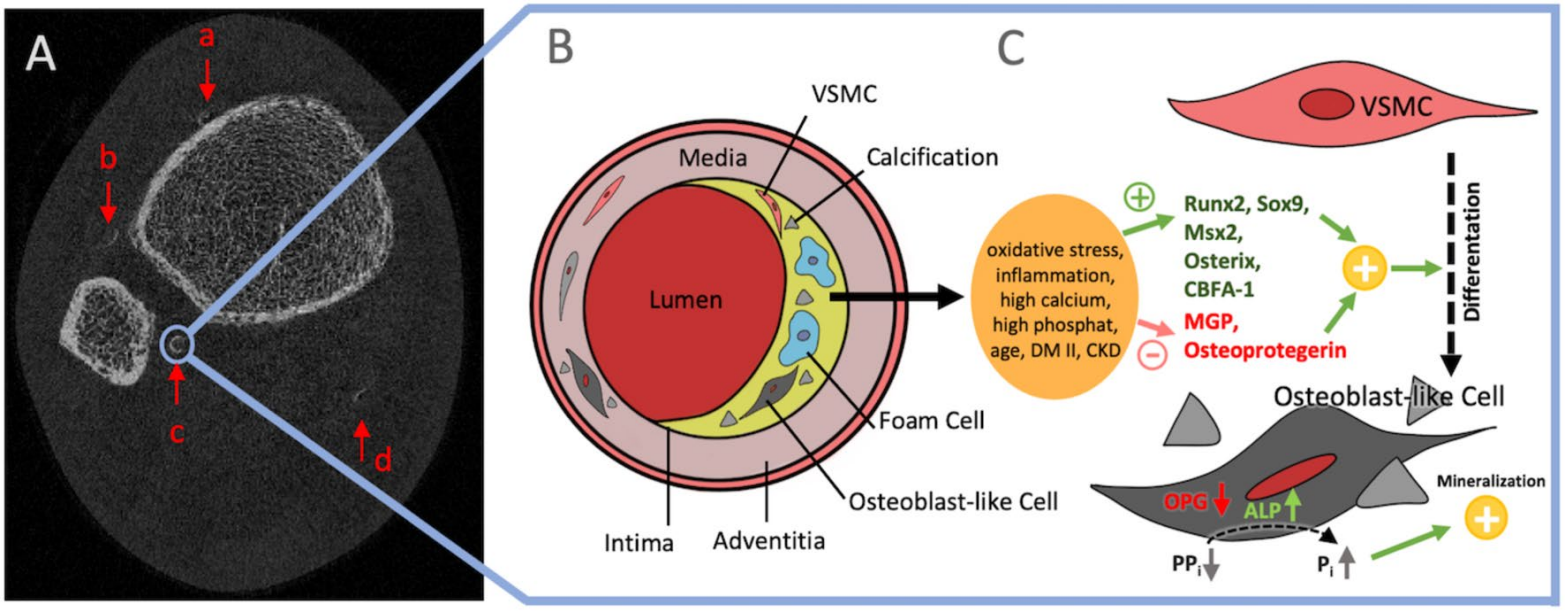
A scheme of the pathophysiology of vascular calcifications with reference to an example of an axial HR-pQCT image of the right lower leg (**A**). Four lower leg arterial calcifications are visible in the anatomical area of the following vessels: **a** anterior tibial artery, **b** branch of fibular artery, **c** fibular artery, **d** posterior tibial artery (**A**). In the blue frame, the calcification process of the vessels is depicted schematically. In the arteriosclerotic plaques, foam cells and VSMC are present and lead to calcifications when the differentiation of VSMC to osteoblast-like cells is triggered (**B**). The differentiation of VSMC into osteoblast-like cells is promoted by stimuli such as inflammation, oxidative stress, high calcium and phosphate levels. Thereby, the expression of Runx2, Sox9, Msx2, Osterix is promoted and transcription of MGP and Osteoprotegerin is inhibited, which promotes osteoblast-like functions in VSMC after differentiation (**C**). Furthermore, enzymes which are associated with calcification in vessels, such as ALP, are activated. *VSMC:* vascular smooth muscle cell, *MGP:* matrix G protein, *ALP:* alkaline phosphatase

Vascular health often arises in discussions between patients and physicians regarding calcium or vitamin D supplementation to treat osteoporosis, due to concerns that calcium supplementation could lead to vascular calcification, such as atherosclerosis or arteriosclerosis. Chowdhury et al. have shown that lower leg arterial calcification (LLAC, Fig. 1A), often detected during bone microarchitecture analysis via HR-pQCT, is a predictor of cardiovascular outcomes [1]. Given that atherosclerosis is associated with strokes and ischemic heart disease [2], which are among the leading causes of death [3], the concerns of patients are understandable. The assumed link between vascular calcifications and bone microarchitecture is comprehensible due to similarities like high radiological density [4], and a collagen based soft matrix [5].

Paradoxically, decreased bone mineral density, rather than increased density, is often linked to greater arterial calcification—an occurrence termed the ‘calcification paradox’ observed in conditions like chronic kidney disease and osteoporosis [6–11]. This paradox questions a link between bone microarchitecture, mineral content, and vascular calcification such as LLAC. The molecular mechanisms of arterial wall calcification as well as the proteins involved, which lead to the deposition of hydroxyapatite, are very similar to bone calcification. The RANK/RANKL/OPG system plays a significant role, a potential explanation of the paradox. TGF-β induces protective OPG and low RANKL levels in bone, but vice versa increases RANKL and lowers OPG in endothelial cells [11]. Low OPG levels in the arterial wall are linked to high levels of alkaline phosphatase, favoring the calcification of soft tissue by supplying inorganic phosphate (P_i_) from inorganic pyrophosphate (PP_i_) for hydroxyapatite formation [12].

The vascular calcification process is multifactorial and influenced by aging, renal failure, hypertension, osteoporosis and others, mainly affecting the media of arteries leading to the transdifferentiation of vascular smooth muscle cells (VSMC) of the artery into bone-like cells (Fig. 1) [11]. Within this process, bone-related transcription factors such as Runx2, Sox9, Msx2, core binding factor α1 (CBFA-1) and osterix are upregulated driving the differentiation of VSMC to osteoblast-like cells [13, 14]. Simultaneously, the local absence of inhibitors such as PP_i_, matrix G protein (MGP) and osteoprotegerin in the vessel wall contributes to the vascular calcification [13] (Fig. 1).

Initial studies on the relationship between vascular calcification and bone microarchitecture reported sex specific results. Paccou et al. explored the interaction between LLAC and bone microarchitecture in the HCS cohort (Hertfordshire Cohorts Study), revealing distinct cortical and trabecular bone alterations in females with LLAC. For males, Paccou et al. did only find alterations in the Tb.N when comparing patients with and without LLAC [6]. Similarly, Guzman et al. found that higher cortical porosity in both sexes and lower cortical bone mineral density in female patients were linked to more advanced coronary artery calcification, suggesting sex-specific bone changes related to vascular calcification [15]. Cejka et al. also observed an inverse relationship between bone microarchitecture and coronary artery calcification in dialysis patients of both sexes [16].

However, given that most research focuses on high-grade CKD patients and CKD [8, 9, 16, 17] (while CKD is known to cause arteriosclerosis), there is a critical need for studies specifically addressing the interaction between LLAC and bone microarchitecture in patients with normal renal function and decreased bone mineral density as a sign of bone metabolism disturbance. This focus will help clarify the role of bone metabolism disturbances in vascular calcification in both females and males.

Therefore, we first hypothesized that patients stratified for LLAC would exhibit decreased trabecular and cortical bone structures, as assessed by cortical thickness and porosity measures, and trabecular morphometric measures using second-generation HR-pQCT. The aim of our study was to investigate the relationship between bone quality (3D bone volume, microarchitecture, and density of the trabecular and cortical compartments) and the volume and density of vascular calcification in patients with low bone density, controlled for kidney function. Additionally, we hypothesized that an increase in plaque density and volume would be associated with reduced bone microarchitecture. Furthermore, we hypothesized that patients with LLAC would show laboratory findings favoring atherosclerosis, such as tendencies towards higher calcium and phosphate levels within the normal range. Considering the known sex dependence of atherosclerosis, we conducted separate examinations for each sex.

## Methods

### Study design

774 adult patients who presented at our outpatient clinic for bone diseases between August 2021 and February 2022 were retrospectively evaluated. As part of routine diagnostics, patients undergo DXA and HR-pQCT imaging as well as laboratory analysis. All patients with detectable lower leg arterial calcifications (LLAC) in the scan volume were included into the LLAC group, whereas patients without detected LLAC were assigned to the control group. As outlined by Paccou et al., arterial calcifications of the lower leg are defined as hyperdensities of linear or tubular character with a circular or semi-circular shape [6]. All HR-pQCT scans were meticulously examined by a single observer, who manually assessed each image to identify LLAC based on anatomical positions, allowing for a distinction between anterior and posterior vessels, as illustrated in Fig. 1A. Patients with genetic diseases, CKD grade ≥ 3B (< 45 ml/min) according to the KDIGO (Kidney Disease: Improving Global Outcomes CKD-MBD Work Group Guidelines [18]) or active tumors were excluded. Calcium supplementation was assessed based on a medical history questionnaire. This study was conducted in accordance with local guidelines and the Declaration of Helsinki with informed consent of participating individuals.

### Dual-Energy X-ray absorptiometry (DXA)

Dual-energy X-ray absorptiometry (Lunar iDXA, GE Healthcare, Madison, WI, USA) scans were performed at the lumbar spine (L1–4) and both proximal femora (femoral neck and total hip). Subsequently, areal bone mineral density (aBMD), T-score and Z-score were determined. For further analysis, the lowest T-score of the lumbar spine and the proximal femora with corresponding aBMD and Z-score were used. Daily calibration scans were performed with the dedicated phantom according to the manufacturer’s recommendations including precision tests following the recommendations of the International Society for Clinical Densitometry (ISCD) [19].

### Biochemical analysis

Blood and Urine samples were analyzed on a routine clinical base at the local laboratory, as described elsewhere [20]. Levels of serum calcium, phosphate, creatinine, alkaline phosphatase (ALP), creatinine, bone-specific alkaline phosphatase (bALP), osteocalcin, parathyroid hormone (PTH) and 25-hydroxyvitamin D (25-OH-D) as well as urinary deoxypyridinoline (DPD) per urinary creatinine were assessed. Glomerular filtration rate (GFR) was estimated by CKD-EPI [21].

### HR-pQCT bone microstructure

All patients were examined with second-generation HR-pQCT (XtremeCT II, Scanco Medical AG, Brüttisellen, Switzerland) at the distal tibia. Scans were carried out according to the manufacturer’s standard in vivo scan protocol (68 kVp, 1470 μA, 43 ms integration time, 60.7 μm voxel size).

Scans at the tibia were taken at a defined offset of 22 mm proximal to the reference line positioned at the inflection point of the endplate of the tibial plafond in accordance with Whittier et al. [22]. The entire scanning volume extended over 168 slices (10.2 mm). Microarchitectural parameters followed the standardized nomenclature of the IOF-ASBMR-ECTS working group and included bone volume to total volume ratio (BV/TV), trabecular number (Tb.N, mm^−1^), trabecular thickness (Tb.Th, mm), trabecular separation (Tb.Sp, mm), cortical thickness (Ct.Th, mm), cortical pore diameter (Ct.Po.Dm, mm) and cortical porosity (Ct.Po). Volumetric bone mineral density was expressed as total BMD (Tt.BMD, mg HA/cm^3^), cortical BMD (Ct.BMD, mg HA/cm^3^), and trabecular BMD (Tb.BMD, mg HA/cm^3^). Geometric values include total bone area (Tt.Ar, mm^2^), trabecular bone area (Tb.Ar, mm^2^), cortical bone area (Ct.Ar, mm^2^), and cortical perimeter (Ct.Pm, mm). All HR-pQCT images were manually examined for motion artifacts [23]. If motion grade 4 or 5 was detected, patients were excluded [22], resulting in the exclusion of 7 patients in total (6.14%).

Previous trauma or microtrauma may influence the presence of calcification and the bone microstructure. Therefore, we manually excluded calcifications that were not associated with vessels, such as heterotopic calcifications, which was the case for 7 patients (6.14%).

### Quantification of LLAC

For quantification of the vessel calcification, XamFlow 1.8.0.0 (Lucid Concepts AG, Zürich, Switzerland) was used. Image stacks were batch loaded and processed via a defined workflow to detect and quantify LLAC in the soft tissue surrounding the bones.

Therefore, first the image stacks were filtered with a gaussian filter (sigma = 3, support = 3) to remove noise. To isolate the full human tissue area from background, a threshold tissue was applied with a lower threshold at −189.8 mg HA/cm^3^ and an upper threshold of 1656.8 mg HA/cm^3^. Subsequently, mineralized tissue was masked using a specified threshold mineral with the lower value set to 169.6 mg HA/cm^3^ and the higher threshold value to 1868.8 mg HA/cm^3^. To address partial volume effects in the atherosclerotic tissue, we chose a relatively high threshold with respect to the density of the atherosclerosis. Next, the bone volume was subtracted from the full tissue volume. Therefore, the largest two elements (fibula and tibia) of the mineralized tissue volumes were chosen to be subtracted from the full tissue volume. The remaining mineral volumes were kept referring to potential arterial calcification.

A despeckling was subsequently added to the workflow to remove small noise artifacts below a volume of 15 voxels. For any mineralized volumes that did not correspond to the region of arteries or if the shape was clearly not arterial wall calcification, we applied a manual correction to exclude these volumes. Mineralized areas (excluding the bones) were than evaluated with respect to density and volume.

### Statistical analysis

Statistical analysis was performed using SPSS software version 28.0.1 (IBM, Armonk, NY, USA), GraphPad Prism 9.5.0 (GraphPad Software, San Diego, CA, USA) and G*Power software version 3.1.9.6 (Heinrich-Heine-University Düsseldorf, Düsseldorf, NRW, Germany). The results are presented as absolute values or as means ± standard deviations. To evaluate normal distribution, the Shapiro–Wilk test was performed. All categorical variables were compared using the Fisher's exact test. For differences between two subgroups, the unpaired two-tailed students *t*-test was used for normally distributed data and the Mann–Whitney-*U* test was performed for non-normally distributed data. Correlation analyses were performed to evaluate the relationships between vascular calcification variables (density and volume) and HR-pQCT bone microstructure and density parameters. For non-normally distributed data Spearmen correlation was used. Additionally, due to interactions among many parameters, a multiple linear regression model (enter method) was used to evaluate the independent predictive value of the various variables on plaque density (mg HA/cm^3^) and plaque volume (# voxels) in lower limb vascular calcifications.

Parameters that showed relevance in previous analyses, such as correlation analysis and (sub-)group comparison were included. In addition to the characteristics of the overall model (R^2^, adjusted R^2^, *F*, and *p*-value), individual regression coefficients (B, *ß*, and *p*-value) were calculated. Variance inflation factors (VIF) were calculated to check for multicollinearities, with no multilinearity indicated if the VIF values were between 1 and 5. We decided to perform propensity score matching in mitigating confounding effects and improving the reliability of our tests [24]. We thereby minimized other influencing factors such as age and BMI between the different groups, enhancing the overall power. However, due to the matching, sample set size and thus calculated power were reduced.

## Results

### Characterization of the study cohort

Of the 774 patients, 114 (63 females and 51 males) exhibited LLAC, representing 14.73%. Given that the occurrence and progression of vascular calcification in atherosclerosis is multifactorial [25], we addressed comparability between cohorts through propensity score matching based on sex, age, and BMI [26]. This method of close matching was employed to minimize substantial demographic differences between patients, thereby enhancing the study's ability to detect specific effects of interest. A total of 132 patients were included (66 in the control group and 66 with LLAC, comprising 39 females and 27 males in each group). The characteristics of our propensity score-matched study cohort are presented in Table 1. The medical history questionnaire was incomplete for 15 female patients (9 without LLAC, 6 with LLAC) and 7 male patients (5 without LLAC, 2 with LLAC).

**Table 1:** Overview of the propensity score-matched study cohort

Parameter	Females without LLAC (n = 39)				Females with LLAC (n = 39)				*p*
	Mean	SD	Min	Max	Mean	SD	Min	Max	
Demographics									
Age (years)	67.3	8.5	48	84	68.8	9.4	52	85	0.467
Weight (kg)	63.7	9.8	49.0	90.3	61.8	9.4	48.3	87.3	0.378
Height (m)	1.65	0.06	1.51	1.77	1.64	0.07	1.51	1.76	0.583
BMI (kg/m^2^)	23.5	3.4	17.8	32.3	23.0	3.1	17.9	30.6	0.507
Calcium supplementation	5 of 30 (16.7%)				11 of 33 (33.3%)				0.156
Arterial hypertension	2 of 39 (5.1%)				5 of 39 (12.8%)				0.431
Diabetes mellitus	0 of 39 (0%)				2 of 39 (5.1%)				0.494
DXA									
Femoral T-score	−2.1	0.9	−3.8	0.0	−2.4	0.9	−3.7	0.4	0.293
Femoral Z-score	−0.7	0.9	−3.0	1.2	−0.9	0.9	−2.7	1.8	0.706
Spinal T-score	−2.4	1.3	−5.1	0.9	−2.6	1.1	−4.4	0.3	0.772
Spinal Z-score	−0.9	1.2	−3.4	2.4	−1.0	1.2	−3.3	1.7	0.882
Normal BMD	5 of 39 (12.8%)				1 of 39 (2.6%)				0.200
Osteopenia (< −1.0)	11 of 39 (28.2%)				12 of 39 (30.8%)				> 0.999
Osteoporosis (≤ −2.5)	23 of 39 (59.0%)				26 of 39 (66.6%)				0.640

Parameter	Males without LLAC (n = 27)				Males with LLAC (n = 27)				*p*
	Mean	SD	Min	Max	Mean	SD	Min	Max	
Demographics									
Age (years)	58.7	10.3	41	83	60.1	11.7	41	83	0.650
Weight (kg)	82.6	14.7	60.0	115.0	82.0	11.6	59.4	106.0	0.877
Height (m)	1.80	0.06	1.70	1.96	1.79	0.07	1.68	1.94	0.339
BMI (kg/m^2^)	25.3	4.5	18.6	36.3	25.6	3.3	20.7	32.4	0.786
Calcium supplementation	3 of 22 (13.6%)				1 of 25 (4.0%)				0.328
Arterial hypertension	0 of 26 (0%)				2 of 26 (7.7%)				0.490
Diabetes mellitus	0 of 27 (0%)				1 of 27 (3.7%)				> 0.999
DXA									
Femoral T-score	−1.4	0.9	−3.0	0.5	−1.4	1.1	−3.4	0.9	0.948
Femoral Z-score	−0.7	1.0	−2.7	1.7	−0.6	1.1	−2.8	1.7	0.838
Spinal T-score	−1.1	1.5	−3.6	1.9	−1.5	1.4	−3.9	1.1	0.426
Spinal Z-score	−0.9	1.5	−3.5	2.0	−1.2	1.4	−3.3	1.7	0.519
Normal BMD	5 of 27 (18.5%)				7 of 27 (25.9%)				0.745
Osteopenia (< −1.0)	16 of 27 (59.3%)				9 of 27 (33.3%)				0.101
Osteoporosis (≤ −2.5)	6 of 27 (22.2%)				11 of 27 (40.7%)				0.241

Females and males were divided according to the presence of LLAC measured by HR-pQCT. In addition, patients were classified into normal areal bone mineral density (aBMD), osteopenia, and osteoporosis based on the T-score measured by DXA. Diabetes mellitus. No significant differences were found between patients with and without LLAC

After propensity score matching for age and BMI the sex-specific group comparisons between patients with and without LLAC showed no differences in age, height, weight, BMI, GFR (CKD-EPI), femoral and spinal T- and Z-score as well as frequency and duration of calcium supplementation, arterial hypertonia and diabetes mellitus, including both diabetes type 1 and 2 (all *p* > 0.05). No differences in the prevalence of osteoporosis (defined by a T-score ≤ -2.5 [27]) was found between females with and without LLAC (LLAC: 66.6% *versus* without LLAC: 59.0%; *p* = 0.482) and males with LLAC and without LLAC (LLAC: 40.7% *versus* without LLAC: 22.2%; *p* = 0.143). In addition, there were no significant differences in received bone-specific therapy between patients with and without LLAC in our groups (Suppl. Table 1). The *post-hoc* power analyses for the t-tests and the correlations resulted in a power < 0.8.

### HR-pQCT bone parameters

**Fig. 2:**
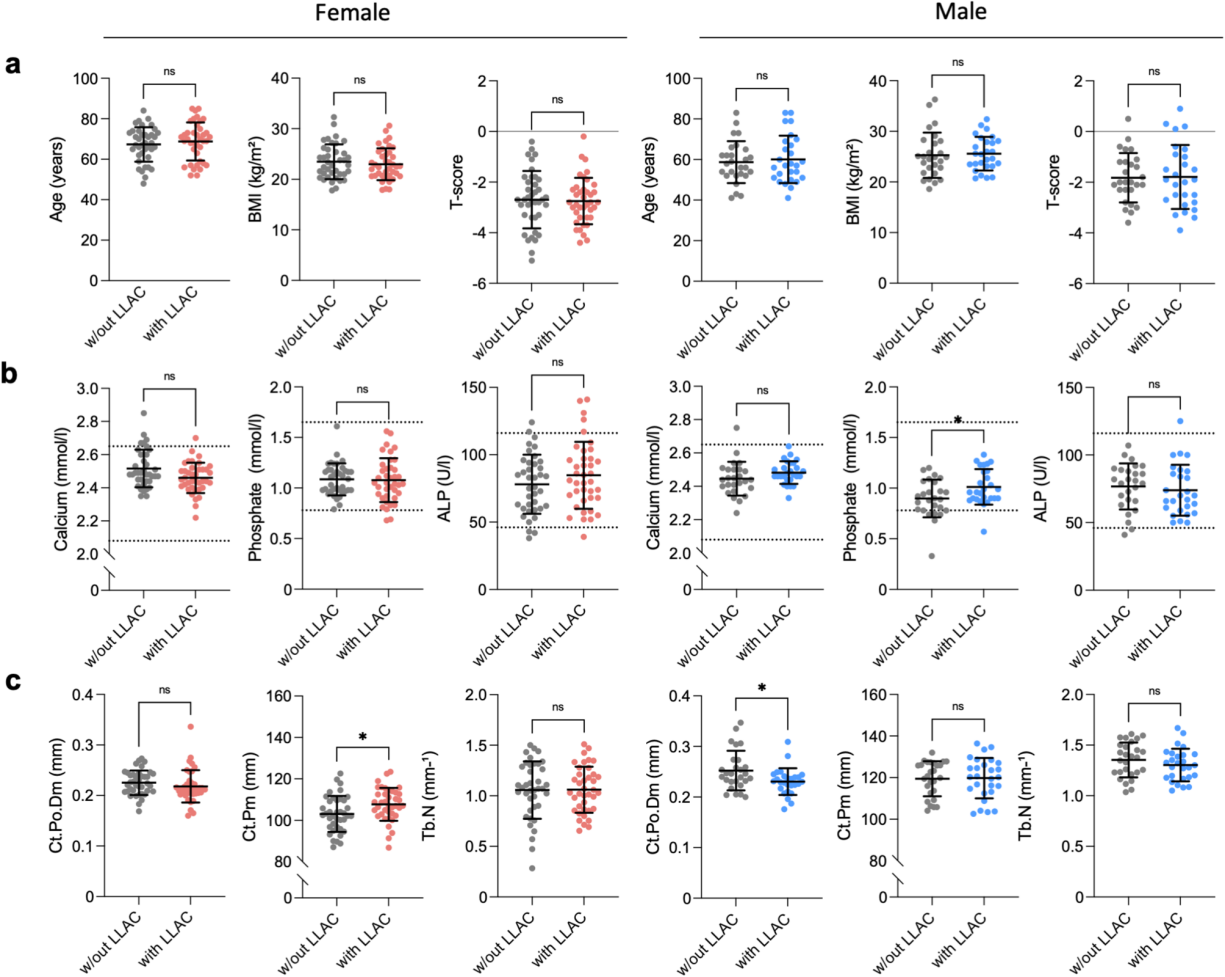
Sex-specific differences between patients with and without LLAC. Comparison of **a** demographic, **b** biochemical, and **c** bone microstructure parameters between patients with and without LLAC. Gray color depicts the control groups without LLAC. Red and blue color represents the female and male cohort with LLAC, respectively. Scatter lines indicate the reference range. *p < 0.05; *ns*  not significant. *BMI* body mass index, *ALP* alkaline phosphatase, *Ct.Po.Dm* cortical pore diameter, *Ct.Pm* cortical perimeter, *Tb.N* trabecular number

Females with LLAC exhibit a significantly higher Ct.Pm compared to females without LLAC (Ct.Pm_with LLAC_ = 107.7 ± 7.9 mm *vs.* Ct.Pm_without LLAC_ = 103.1 ± 8.7 mm; *p* = 0.016) (Fig. 2). Other HR-pQCT parameters did not differ in females. In contrast, males with LLAC exhibit significantly lower Ct.Po.Dm compared with males without LLAC (Ct.Po.Dm _with LLAC_ = 0.230 ± 0.026 mm *vs.* Ct.Po.Dm_without LLAC_ = 0.252 ± 0.039 mm; *p* < 0.027) but did not present any other differences comparing control and LLAC group. (Fig. 2, Table 2).

**Table 2:** HR-pQCT and biochemical parameters of patients with and without LLAC

Parameter	females w/out LLAC		females with LLAC		*p*	males w/out LLAC		males with LLAC		*p*
	Mean	SD	Mean	SD		Mean	SD	Mean	SD	
HR-pQCT										
Tt.BMD (mg HA/cm^3^)	213.9	53.9	195.7	41.4	*0.133*	294.0	48.5	281.9	65.8	*0.448*
Tt.Ar (mm^2^)	703.3	117.6	746.8	109.9	*0.096*	921.9	120.2	926.5	143.2	*0.966*
Tb.BMD (mg HA/cm^3^)	121.8	37.1	118.0	31.4	*0.634*	185.5	37.8	178.7	41.0	*0.528*
BV/TV	0.192	0.046	0.187	0.039	*0.638*	0.273	0.052	0.264	0.056	*0.551*
Tb.N (mm^−1^)	1.057	0.284	1.060	0.228	*0.953*	1.353	0.172	1.303	0.161	*0.277*
Tb.Th (mm)	0.254	0.020	0.249	0.019	*0.283*	0.268	0.026	0.0273	0.023	*0.543*
Tb.Sp (mm)	1.059	0.553	0.979	0.246	*0.768*	0.710	0.110	0.741	0.105	*0.303*
Tb.Ar (mm^2^)	611.8	122.5	663.1	116.2	*0.062*	775.7	125.8	786.5	154.7	*0.780*
Ct.BMD (mg HA/cm^3^)	780.6	64.6	747.0	81.4	*0.315*	837.4	52.3	816.7	64.3	*0.199*
Ct.Th (mm)	1.139	0.298	1.019	0.242	*0.053*	1.527	0.243	1.467	0.342	*0.462*
Ct.Po	0.037	0.016	0.035	0.014	*0.512*	0.035	0.010	0.033	0.013	*0.533*
Ct.Po.Dm (mm)	0.225	0.024	0.218	0.032	*0.117*	0.252	0.039	0.230	0.026	***0.027***
Ct.Pm (mm)	103.1	8.7	107.7	7.9	***0.016***	119.5	8.4	119.8	9.7	*0.898*
Ct.Ar (mm^2^)	96.9	23.3	89.3	18.4	*0.113*	152.4	22.0	146.3	26.2	*0.354*
Laboratory bone parameter										
Calcium (mmol/l)	2.52	0.11	2.46	0.09	*0.061*	2.45	0.10	2.48	0.07	*0.124*
Phosphate (mmol/l)	1.09	0.16	1.08	0.22	*0.862*	0.90	0.19	1.01	0.18	***0.025***
Creatinine mg/dl	1.04	1.49	0.81	0.17	*0.972*	1.03	0.28	0.96	0.18	*0.262*
GFR (CKD-EPI) (ml/min)	77	17	76	15	*0.596*	82	18	85	14	*0.581*
ALP (U/l)	77	22	85	25	*0.216*	77	17	74	19	*0.577*
Osteocalcin (µg/l)	20.9	6.8	22.1	9.2	*0.689*	16.0	7.4	16.7	4.3	*0.680*
25(OH)D (µg/l)	35.9	12.4	37.0	11.6	*0.732*	24.6	13.2	26.4	14.1	*0.563*
b-ALP (µg/l)	12.4	5.0	13.6	5.1	*0.350*	12.1	4.4	10.8	3.8	*0.245*
PTH (ng/l)	50.7	28.0	58.2	30.0	*0.102*	56.7	42.4	48.9	17.4	*0.929*
DPD/Crea (nmol/mmol)	8	2	8	3	*0.197*	6	2	6	2	*0.569*

Comparison of the bone parameters measured by HR-pQCT and biochemical parameters divided by sex and the groups with and without LLAC. Groups were compared using a unpaired two-tailed students t-test for normally distributed data and the Mann–Whitney-U test for non-normally distributed data

*Tt.BMD* total BMD, *HA* hydroxyapatite, *Tt.Ar* total area, *Tb.BMD* trabecular BMD, *BV/TV* bone volume to tissue volume, *Tb.N* trabecular number, *Tb.Th* trabecular thickness, *Tb.Sp* trabecular separation, *Tb.Ar* trabecular area, *Ct.BMD* cortical BMD, *Ct.Th* cortical thickness, *Ct.Po* cortical porosity (pore volume to total volume ratio), *Ct.Po.Dm* cortical pore diameter, *Ct.Pm* cortical perimeter, *Ct.Ar* cortical area, *GFR (CKD-EPI)* glomerular filtrate rate using the equation of chronic kidney disease epidemiology collaboration, *ALP* alkaline phosphatase, *25-OH-D* 25-hydroxyvitamin D, *b-ALP* bone-specific alkaline phosphatase, *PTH* parathyroid hormone, *DPD/Crea* deoxypyridinoline per creatinine in the urine

### Laboratory serum and urine-parameters in patients with and without LLAC

When comparing the laboratory parameters of females with and without LLAC, no significant differences were found (Fig. 2, Table 2). However, in males with LLAC a significantly higher mean serum phosphate level was found, compared to males without LLAC (P_LLAC_ = 1.01 ± 0.18 mmol/l *vs.* P_without LLAC_ = 0.90 ± 0.19 mmol/l; *p* = 0.025). This was the only significant difference that could be found for laboratory bone parameters in both sexes.

### Correlation of LLAC with demographic parameters, bone microarchitecture and density in the distal tibia

Upon evaluating linear correlations between demographic parameters and LLAC appearance, we found no significant associations between LLAC density or volume and age, BMI, T-score, or Z-score. This lack of association was consistent across both sexes.

In females, HR-pQCT imaging revealed no significant correlations between the parameters and LLAC density or volume. In contrast, in males, age was positively correlated with LLAC density (r_rho_ = 0.452; *p* = 0.020), suggesting that older age may be associated with increased vascular calcification density. Additionally, a negative correlation was observed between trabecular number (Tb.N) and LLAC density (r_rho_ = -0.404; *p* = 0.041), indicating that lower trabecular number is associated with higher vascular calcification density. These were the only significant correlations in males for both, vessel calcification density and volume (Table 3).

**Table 3:** Associations between bone microarchitecture and density as well as volume of LLAC using Spearman’s correlation

Parameter	Females				Males			
	Density (mg HA/cm3)		Volume (# voxel)		Density (mg HA/cm3)		Volume (# voxel)	
	r_rho_	*p*	r_rho_	*p*	r_rho_	*p*	r_rho_	*p*
HR-pQCT								
Tt.BMD (mg HA/cm^3^)	−0.156	0.341	−0.245	0.133	−0.026	0.901	0.068	0.741
Tt.Ar (mm^2^)	0.095	0.566	0.047	0.776	0.003	0.988	0.134	0.515
Tb.BMD (mg HA/cm^3^)	−0.091	0.580	−0.180	0.272	−0.039	0.851	0.086	0.677
BV/TV	−0.063	0.703	−0.131	0.425	−0.004	0.985	0.174	0.396
Tb.N (mm^−1^)	−0.149	0.364	−0.162	0.326	−0.404	**0.041**	−0.111	0.589
Tb.Th (mm)	−0.201	0.220	0.128	0.437	−0.199	0.330	0.093	0.652
Tb.Sp (mm)	0.117	0.477	0.176	0.284	0.291	0.149	0.018	0.930
Tb.Ar (mm^2^)	0.103	0.533	0.083	0.615	0.032	0.875	0.192	0.346
Ct.BMD (mg HA/cm^3^)	−0.102	0.536	−0.269	0.098	−0.110	0.594	−0.048	0.818
Ct.Th (mm)	−0.078	0.636	0.070	0.670	−0.052	0.802	−0.029	0.888
Ct.Po	0.266	0.101	0.247	0.130	0.150	0.464	−0.087	0.672
Ct.Po.Dm (mm)	0.070	0.674	−0.007	0.964	0.289	0.152	−0.147	0.473
Ct.Pm (mm)	0.021	0.898	0.110	0.505	0.024	0.906	0.221	0.278
Ct.Ar (mm^2^)	−0.133	0.421	0.012	0.940	−0.104	0.615	0.058	0.777

There was no significant correlation between any bone parameter and plaque volume (# voxel)

The significant p−value (p < 0.05) is indicated in bold

*Tt.BMD* total BMD, *HA* hydroxyapatite, *Tt.Ar* total area, *Tb.BMD* trabecular BMD, *BV/TV* bone volume to tissue volume, *Tb.N* trabecular number, *Tb.Th* trabecular thickness, *Tb.Sp* trabecular separation, *Tb.Ar* trabecular area, *Ct.BMD* cortical BMD, *Ct.Th* cortical thickness, *Ct.Po* cortical porosity (pore volume to total volume ratio), *Ct.Po.Dm* cortical pore diameter, *Ct.Pm* cortical perimeter, *Ct.Ar* cortical area

### Multiple linear regression of bone parameters and LLAC

In females, age positively influenced LLAC density, while Tb.Th negatively predicted it (Table 4). No significant model for atherosclerosis volume was found in females indicating no interaction of LLAC volume and the examined parameters.

**Table 4:** Multiple linear regression models for analysis of independent predictors of plaque density (mg HA/cm^3^) and plaque volume (# voxel)

Parameter	Females			Males		
	B	ß	*p*	B	ß	*p*
Plaque density (mg HA/cm^3^)						
Constant	441.750		< 0.001	429.039		0.103
Age (years)	2.156	0.424	**0.011**	2.274	0.459	**0.024**
Tt.BMD (mg HA/cm^3^)	0.021	0.018	0.936	0.258	0.293	0.534
Ct.Po.Dm (mm)	424.521	0.284	0.174	799.395	0.364	0.258
Tb.N (mm^−1^)	−41.935	−0.200	0.263	−126.386	−0.351	0.206
Tb.Th (mm)	−1016.308	−0.411	**0.029**	−948.706	−0.378	0.161
	R^2^ = 0.332			R^2^ = 0.587		
	R^2^ adjusted = 0.231			R^2^ adjusted = 0.344		
	F (5, 33) = 3.279, *p* = **0.016**			F (5, 21) = 2.202, *p* = 0.093		
Constant	−7003.194		0.867	282,278.473		0.190
Age (years)	−125.514	−0.083	0.657	1419.198	0.321	0.081
Tt.BMD (mg HA/cm^3^)	−149.744	−0.435	0.110	895.203	1.138	**0.015**
Ct.Po.Dm (mm)	39,256.284	0.088	0.714	−1.323E-6	−0.674	**0.030**
Tb.N (mm^−1^)	10,626.383	0.170	0.413	−242,613.593	−0.754	**0.006**
Tb.Th (mm)	143,381.604	0.195	0.361	110,729.224	0.050	0.840
	R^2^ = 0.299			R^2^ = 0.660		
	R^2^ adjusted = 0.089			R^2^ adjusted = 0.435		
	F (5, 33) = 0.647, *p* = 0.665			F (5, 21) = 3.235, *p* = **0.026**		

Significant *p*-values (< 0.05) are indicated in bold

*HA* hydroxyapatite, *Tt.BMD* total BMD, *Ct.Po.Dm* cortical pore diameter, *Tb.N* trabecular number, *Tb.Th* trabecular thickness

In males, the multiple linear regression model showed that higher Tt.BMD positively predicted LLAC volume, while increased Ct.Po.Dm and Tb.N negatively impacted LLAC volume. This indicates that lower bone density and decreased bone microarchitecture are associated with increased LLAC volume. In contrast to LLAC density in females, age could not be confirmed as an independent predictor for LLAC volume in males in our cohort. No multicollinearities were found among the predictors. The *post-hoc* power analyses for the linear regression models revealed consistent power > 0.9.

## Discussion

This study aimed to explore the relationship between bone health and vascular calcification in male and female patients with reduced bone mineral density and adequate renal function. Our findings indicate that in females, bone parameters are primarily coupled with LLAC density, whereas in males LLAC volume is more significantly associated. These results highlight the pattern of interaction between bone microarchitecture and vascular calcification in different sexes.

Due to our cohort primarily including patients with reduced aBMD (osteopenia or osteoporosis), we examined more females, despite vascular calcifications being more common in males similar to Nielsen et al. when studying hypoparathyroidism [10]. We excluded patients with high-grade chronic kidney disease (CKD) but included those with low bone mineral density (BMD), as initial HR-pQCT studies have linked reduced bone microarchitecture to increased coronary artery calcification (CAC) [15, 28] and lower leg arterial calcification (LLAC) [6, 8]. Previous studies have primarily focused on cohorts with advanced CKD, which is associated with significant bone loss. This condition also leads to an imbalance in electrolyte homeostasis, hampering bone mineralization and promoting vascular calcification [8, 9, 16]. However, no studies have specifically investigated patients with low aBMD without end-stage CKD.

In our cohort, the frequency and duration of calcium supplementation were similar between patients with and without LLAC in both sexes, aligning with literature and epidemiological data that show no association between calcium intake and cardiovascular mortality [7, 29]. Yet, focusing on blood serum levels, in males with LLAC, phosphate was higher compared to males without LLAC, a common observation in patients with atherosclerosis [30]. In line, human VSMC undergo vesicle-mediated calcification in response to high phosphate levels [31, 32], which emphasizes the importance of electrolyte homeostasis. Interestingly, T-scores between the groups did not differ, suggesting that factors beyond bone mineral density, such as bones three-dimensional structure, may be linked to the frequency of LLAC.

Three-dimensional HR-pQCT revealed that females with LLAC exhibited increased Ct.Pm in the distal tibia, indicating a potential acceleration of biological age by the typical rise of Ct.Pm with age [33]. In line, Paccou et al. have shown that females with LLAC have significantly decreased cortical area and thickness [6]. In males with LLAC, the smaller cortical pore diameter may result from microvascular bone tissue calcification; however, this parameter is limited to detecting pores only within the resolution's limits and those that do not intersect the periosteal or endocortical surfaces. A connection of Ct. Po and arterial calcification was also found by Guzman et al. highlighting the importance of the parameter for future studies [15]. Furthermore, Ct.Po and Ct.Po.Dm are known to have a low reproducibility and exhibited a high variability, creating uncertainties with respect to this parameter [34]. In general, variables of variation and LSC (Least Significant Change) have to be taken into account when considering changes measured by HR-pQCT [35].

No interactions in the two-parameter correlation analysis between HR-pQCT parameters and LLAC parameters were observed in females (Table 3). This may be due to parameter interactions, which can be accounted for using multiple linear regression. This particular interaction can be seen in males, where LLAC density increases with age and decreases with Tb.N. Similarly, Tb.N interactions were seen by Paccou et al. for males as the only variable differing between patients with and without LLAC [6]. However, Tb.N is known to decrease with age [36], pointing to the well-known association of atherosclerosis and age [37].

Accounting for parameter interactions, the multiple linear regression analysis revealed that in females, bone loss related [38] trabecular thinning [39, 40] positively correlates with LLAC densification. These results are in line with Paccou et al. [6]. However, bone resorption was not elevated in females with LLAC compared to those without LLAC at the time point of HR-pQCT (Table 2), suggesting that bone resorption has already occurred or may be on a chronic but low level. Noteworthy, no significant predictors were found for the volume of LLAC in females.

No significant associations were found between plaque density and LLAC in males. However, multiple linear regression indicates that increasing LLAC volume is associated with decreased bone microarchitecture and mass, while the remaining tissue exhibits higher mineralization. Guzman et al. showed similar trends with coronary artery calcification, with more pronounced structural and density changes in males than females. [15]. Thus, increased bone loss and transient changes [11] in calcium and phosphate metabolism may lead to higher arterial calcification in males, emphasizing the need of controlled calcium and bone homeostasis in both sexes. Comparing the sex-specific differences in LLAC it stands out that females have an association with density, compared to males with volume. Increased LLAC volume may be more likely to cause severe arterial injury or obstruction than merely increasing the density of existing LLAC. This underscores the importance of considering sex-specific patterns in arterial calcifications [41].

The strengths of this study lies in its focus on a bone disease-related cohort without significant kidney function impairment, which minimizes confounding effects on skeletal and vascular health. We performed extensive propensity score matching in order to minimize the confounding by multiple influencing factors such as age or BMI. While propensity score matching results is a relatively small sample size, discrimination for pairwise testing is increased due to a reduction of confounding factors harmonizing the compared cohorts. Nonetheless, our study was limited to a European cohort of mostly Caucasian individuals. In the context of calcium supplementation, this variable was only dichotomous, however, supplementation was patient specifically tailored according to PTH levels which did not differ between groups.

In conclusion we found no association between LLAC and calcium supplementation. Our findings suggest a potential sex-specific pattern in LLAC manifestation, with increasing LLAC density observed in females and increasing LLAC volume in males. These changes are associated with changes in age and Tb.Th in females and Tt.BMD, Ct.Po.Dm and Tb.N in males. Taken together, our results suggest a potential link between vascular calcification and reduced bone density in patients with low aBMD without renal failure. Our findings indicate the significance of rebalancing impaired bone metabolism to protect against bone damage and prevent arterial calcification accumulation.

## Supplementary Information

Below is the link to the electronic supplementary material.

Supplementary file1 (DOCX 13011 KB)
