# Supplementary material for: Sex-Specific Association Patterns of Bone Microstructure and Lower Leg Arterial Calcification
Source: Calcif Tissue Int. 2024 Oct 14;115(5):636–47. doi: 10.1007/s00223-024-01299-w (PMC11531430; doi:10.1007/s00223-024-01299-w)
Supplement: Supplementary file 1 — Supplementary file1 (DOCX 13011 KB) [file 223_2024_1299_MOESM1_ESM.docx]

**Supplementary Material**

**Sex-specific association patterns of bone microstructure and lower leg arterial calcification favoring plaque density in women and plaque volume in men**


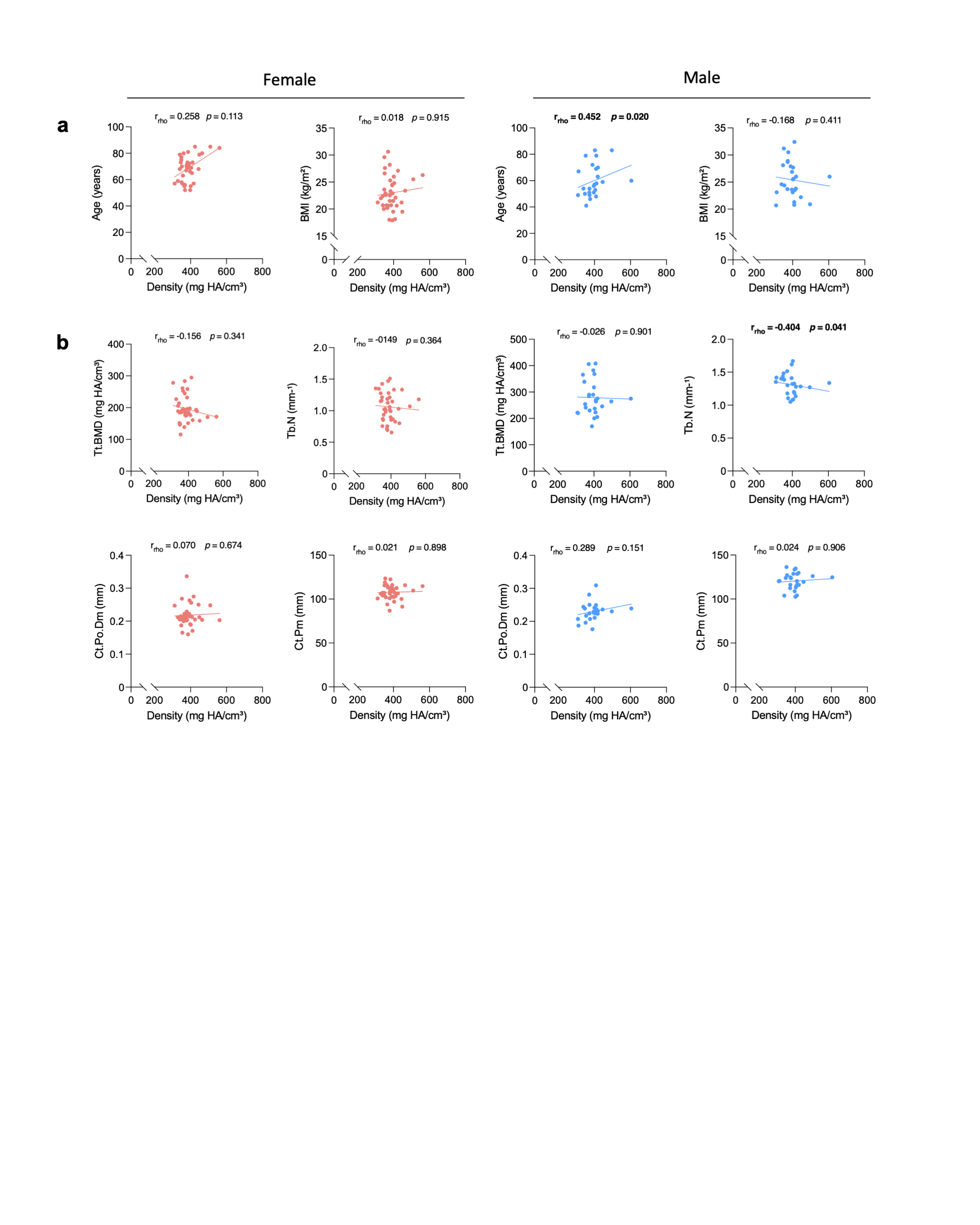
Mikolaj Bartosik, Alexander Simon, Björn Busse, Florian Barvencik, Michael Amling, Ralf Oheim, Felix N. von Brackel

**Supplementary Figure 1 - Spearman’s correlation analysis for LLAC density**. Correlation analysis of (a) age and BMI as well as (b) bone microstructure parameters with the LLAC density. Significant p-values (< 0.05) and corresponding r_rho_-values are indicated in bold. BMI: body mass index; Tt.BMD: total BMD; Tb.N: trabecular number; Ct.Po.Dm: cortical pore diameter; Ct.Pm: cortical perimeter.

|  |  |  | **females w/out LLAC** (n = 39) | **females with LLAC** (n = 39) |  |
| --- | --- | --- | --- | --- | --- |
| Bone specific medication | | | Frequency | Frequency | ***p*** |
|  | Bisphosphonates | | 5 of 39 (12.8%) | 6 of 39 (15.4%) | >0.999 |
|  | Teriparatid | | 1 of 39 (2.6%) | 0 of 39 (0.0%) | >0.999 |
|  | Denosumab | | 6 of 39 (15.4%) | 9 of 39 (23.1%) | 0.567 |
|  | Romosozumab | | 2 of 39 (5.1%) | 3 of 39 (7.7%) | >0.999 |
|  | Basistherapy | | 25 of 39 (64.1%) | 21 of 39 (53.8%) | 0.490 |
|  |  |  | **males w/out LLAC** (n = 27) | **males with LLAC** (n = 27) |  |
|  | Bisphosphonates | | 1 of 25 (4.0%) | 1 of 26 (3.8%) | >0.999 |
|  | Denosumab | | 1 of 25 (4.0%) | 1 of 26 (3.8%) | >0.999 |
|  | Basistherapy | | 23 of 25 (92.0%) | 24 of 26 (92.3%) | >0.999 |

**Supplementary Table 1 - Overview of bone-specific medication in patients with and without LLAC.**

Women and men were classified according to the presence of LLAC measured by HR-pQCT. The proportions of patients per group are presented in absolute and relative terms.
